# Rapid decline in visceral adipose tissue over 1 month is associated with poor prognosis in patients with unresectable pancreatic cancer

**DOI:** 10.1002/cam4.3964

**Published:** 2021-05-16

**Authors:** Oki Nakano, Hirokazu Kawai, Takamasa Kobayashi, Junji Kohisa, Satoshi Ikarashi, Kazunao Hayashi, Junji Yokoyama, Shuji Terai

**Affiliations:** ^1^ Division of Gastroenterology and Hepatology Graduate School of Medical and Dental Sciences Niigata University Niigata Japan; ^2^ Department of Internal Medicine Nagaoka Chuo General Hospital Nagaoka Japan; ^3^ Department of Internal Medicine Niigata Prefectural Shibata Hospital Shibata Japan; ^4^ Department of Gastroenterology Nagaoka Red Cross Hospital Nagaoka Japan

**Keywords:** pancreatic cancer, sarcopenia, subcutaneous adipose tissue, visceral adipose tissue

## Abstract

**Background:**

Involuntary weight loss related to cachexia is common in patients with advanced cancer, but the association between body composition changes and survival is still unclear in pancreatic cancer.

**Methods:**

We retrospectively reviewed the clinical outcomes of 55 patients with advanced pancreatic cancer undergoing palliative therapy or best supportive care (BSC). The skeletal muscle index (SMI), visceral adipose tissue index (VATI), subcutaneous adipose tissue index (SATI), and visceral to subcutaneous adipose tissue area ratio (VSR) were calculated based on the cross‐sectional area on two sets of computed tomography images obtained at cancer diagnosis and 1 month later before treatment. The prognostic value of body composition indexes at diagnosis and the changes in those indexes over 1 month was then evaluated.

**Results:**

In total, 45 patients (81.8%) received chemotherapy, chemoradiation, or radiation therapy, whereas the remaining patients underwent BSC. There were 27 patients (49.1%) who had low SMI at cancer diagnosis. Univariate analysis showed no significant associations between the baseline body composition indexes including SMI, VATI, SATI, and VSR and survival. Meanwhile, male sex (HR, 2.79; 95% CI, 1.16–6.71, *p* = 0.022) and higher decrease in VATI over 1 month (HR, 2.41; 95% CI, 1.13–5.13, *p* = 0.023) were identified as independent risk factors for mortality in multivariate analysis.

**Conclusion:**

Rapid decline in VAT over 1 month is closely associated with poorer survival in unresectable advanced pancreatic cancer. A short‐term assessment of body composition changes may be a rational approach to predict prognosis in these patients.

## Graphical abstract

We evaluated the prognostic impact of body composition changes in patients with unresectable pancreatic cancer. We revealed that rapid decline in vsceral adipose tissue over 1 month is closely associated with poorer survival in those patients.

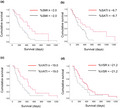

## 1 INTRODUCTION

Pancreatic cancer is the fourth leading cause of cancer‐related mortality worldwide.^1^ Despite efforts to improve survival rates by developing newer treatment methods including systemic chemotherapy and surgery, the prognosis of patients with pancreatic cancer remains poor. The 5‐year survival rate is only 9% at all cancer stages and 3% at the advanced stage with distant metastasis.^1^ One reason for the high mortality is that patients frequently have unresectable cancer at diagnosis, limiting the potential for curative treatment.

Emerging studies have shown close associations between body composition and survival in cancer patients. Pancreatic cancer patients frequently experience involuntary body weight loss related to cachexia along with cancer progression. Of the body composition changes, skeletal muscle (SM) depletion, which is a major feature of sarcopenia, is recognized as a risk factor for mortality in some malignancies including pancreatic cancer.^2^, ^3^, ^4^, ^5^ However, van Dijk reported no association between low SM mass and survival in pancreatic cancer.^6^

Body fat primarily consists of subcutaneous adipose tissue (SAT) and visceral adipose tissue (VAT), which are not only located in two distinct compartments, but also have different metabolic activities and impact on disease progression.^7^ Meanwhile, the prognostic value of SAT and VAT remains a subject of debate in many types of cancer.^3^, ^4^, ^6^, ^8^, ^9^, ^10^, ^11^, ^12^

Intriguingly, recent studies demonstrated that the body composition changes during 3 months or longer from diagnosis is an independent predictor of mortality in patients with pancreatic cancer.^2^, ^13^ However, predicting prognosis in such a long evaluation period would not be beneficial in pancreatic cancer patients who have poor survival. A brief‐time assessment for prognosis is required for those patients.

This study aimed to elucidate the prognostic significance of the body composition changes over 1 month from diagnosis in patients with inoperable pancreatic cancer. Toward this goal, we measured the cross‐sectional areas of SM, SAT, and VAT on computed tomography (CT) images obtained at the time of cancer diagnosis and 1 month later in patients with unresectable advanced pancreatic cancer and analyzed their association with patient characteristics and overall survival (OS).

## 2 METHODS

### 2.1 Study design and patients

This retrospective study was approved by the ethics committee of theNiigata University School of Medicine (approval number 2442) and was conducted in accordance with the 1975 Helsinki Declaration and its later amendments. The requirement for additional informed consent to participate in this study was waived owing to the use of anonymized data.

We reviewed the clinical records of patients with unresectable advanced pancreatic cancer who were treated with chemotherapy, chemoradiation therapy (CRT), radiation therapy (RT), or best supportive care (BSC) at Niigata University Hospital between January 2005 and May 2016. Among them, 55 patients who underwent CT examinations twice, with an approximate 1‐month interval (15–59 days), before treatment were eligible for this study. In most cases, the first CT examination was performed for the diagnosis of the cancer, and the second was implemented for evaluation of the cancer extension. Patients who underwent curative resection or concurrently had other advanced malignancies were excluded. Advanced pancreatic cancer was diagnosed based on CT images, magnetic resonance imaging, and/or histopathological examinations. The patients were classified based on their TNM stage, according to the guidelines proposed by the Japan Pancreas Society (JPS),^14^ which has subtle differences in tumor extent of T4 and N1 from the UICC staging systems.^15^

### 2.2 Body composition analysis

**FIGURE 1:**
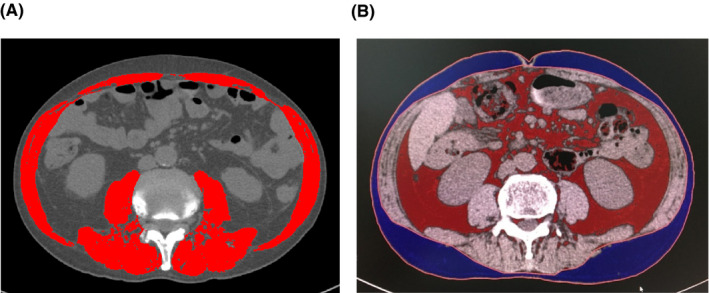
CT images for measurement of body composition. (A) The skeletal muscle area at the third lumbar vertebra level is highlighted in red. (B) The subcutaneous adipose tissue area is highlighted in blue and the visceral adipose tissue area is in red

Body mass index (BMI, kg/m^2^) was calculated by dividing weight (kg) with height squared (m^2^). To assess body composition mass, cross‐sectional CT images were used to measure the body composition area: SM area (cm^2^) was determined at the third lumbar vertebra (L3) level by using SliceOmatic software version 5.0 (Tomovision, Montreal, QC, Canada). Meanwhile, both SAT and VAT area (cm^2^) were measured at the umbilical level by using Ziostation2 (Ziosoft, Inc., Japan). As shown in Figure 1, the software can segment tissue boundaries depending on CT Hounsfield Units (HU) thresholds. The ranges of the HU thresholds for each tissue were within −29 to +150 HU for SM, −190 to −30 for SAT, and −50 to −150 for VAT.^16^ SM area at the L3 level was normalized using the square of the height as SM index (SMI, cm^2^/m^2^). Similarly, SAT and VAT areas (cm^2^) were normalized as subcutaneous adipose tissue index (SATI, cm^2^/m^2^) and visceral adipose tissue index (VATI, cm^2^/m^2^). In addition, the VAT to SAT area ratio (VSR) was calculated to assess the distribution of abdominal adipose tissue. To obtain the standardized percentage changes in each body composition index over 30 days, the percentage changes in SMI, SATI, VATI, and VSR were divided by the interval days between the first and the second CT examinations and compensatively multiplied by 30 (%SMI, %SATI, %VATI, and %VSR, respectively). The cutoff values of low SMI, which indicates depletion of SM mass, was defined as less than 42.2 (cm^2^/m^2^) for males and less than 33.9 (cm^2^/m^2^) for females according to a previous study of Asian subjects of unresectable pancreatic cancer treated with palliative chemotherapy.^2^

### 2.3 Statistical analysis

Survival was counted from the date of CT examination at initial diagnosis of pancreatic cancer to the date of death or end of follow‐up. ​Overall survival (OS​) was estimated using the Kaplan–Meier method, and survival curves were compared using the log‐rank test. Baseline characteristics, body composition indexes, and their percentage changes were calculated as medians with 95% confidence intervals (CI). Baseline characteristics were evaluated using the Mann‐Whitney U test for continuous variables and using the Fisher's exact test or Pearson's chi‐square test for categorical variables, as appropriate. Spearman's rank correlation coefficients were used to analyze the correlation among body composition variables. The potential prognostic factors for OS identified in univariate analyses were included in the multivariate analyses using the Cox proportional hazards model. The optimum cutoff values for body composition indexes were determined by minimizing *P* values when there was a significant difference in log‐rank test, as previously reported.^17^ Alternately, the medians of the variables were used as the cutoff values unless there was a significant difference. All statistical analyses were performed using SPSS version 21.0 (SPSS Japan Inc., Tokyo, Japan). A *p* value of < 0.05 was considered statistically significant.

## 3 RESULTS

### 3.1 Baseline characteristics and changes in body composition indexes

In total, 42 (76.4%) of the patients were male. The median age was 67 years (range, 35–85 years), and the median OS was 277 days (range, 31–1,236 days). The baseline characteristics and the rates of change in body composition indexes over 1 month according to sex are shown in Table 1. There were 35 (63.6%) patients with stage IVb disease due to distant metastasis at the time of diagnosis. The primary tumor locations in the pancreas were the head in 33 (60.0%) patients and the body or tail in 22 (40.0%). Biliary obstruction developed in 19 (34.5%) patients, and 9 (16.4%) patients underwent biliary bypass or gastrointestinal surgery. There was no significant difference in OS between patients with or without biliary obstruction, or between patients receiving surgery or conservative treatment, by log‐rank test (data not shown). With respect to treatment, 34 patients were treated with chemotherapy, 10 with CRT, 1 with RT, and 10 received BSC. The regimens of the first‐line chemotherapy were as follows: FOLFIRINOX in 3 patients, gemcitabine‐based combination with S‐1, CDDP, nab‐paclitaxel, or erlotinib in 17 patients, gemcitabine monotherapy in 13 patients, and S‐1 monotherapy in 1 patient. The RT was performed at a maximum dose of 50.4 Gy in 28 fractions focused on the locally advanced cancer area. In patients who underwent CRT, the following regimens were administered during RT: gemcitabine +CDDP + S‐1 in 1 patient, gemcitabine monotherapy in 3 patients, and S‐1 monotherapy in 6 patients. With regard to body composition indexes at diagnosis, the median BMI was 21.2 kg/m^2^ (range, 14.1–28.1 kg/m^2^), and 27 patients (49.1%) had low SMI.

**TABLE 1:** Baseline patient characteristics

Characteristics	Total (n = 55)	Male (n = 42)	Female (n = 13)	*p* value
Age (years)	67 [35–85]	68 [44–85]	66 [35–76]	0.341
Observation period (days)	277 [31–1236]	215 [31–1002]	385 [171–1236]	0.029
Cancer stage (I / II / III / IVa / IVb)	0 / 1 / 3 / 16 / 35	0 /1 / 3 / 9 / 29	0 / 0 / 0 / 7 / 6	0.122
Primary localization (head/body or tail)	33/22	22 / 14	5 / 8	0.700
Biliary obstruction (yes)	19	15	4	0.743
Surgical operation (yes)	9	8	1	0.533
gastrojejunal bypass	3	3	1	
choledochojejunostomy	3	3	0	
enterostomy	1	1	0	
colostomy	1	0	1	
gastrojejunal bypass and choledochojejunostomy	1	1	0	
Chemotherapy / CRT or RT/BSC	34 / 11 / 10	25 / 8 / 9	9 / 3 / 1	0.421
Albumin (g/dl)	3.9 [1.7–4.9]	3.9 [1.7–4.9]	3.9 [2.5–4.7]	0.889
BMI (kg/m^2^)	21.2 [14.1–28.1]	21.0 [16.2–27.4]	21.7 [14.1–28.1]	0.322
SMI (cm^2^/m^2^)	40.1 [28.3–59.5]	42.1 [28.3–59.5]	35.2 [29.2–50.3]	0.004
SATI (cm^2^/m^2^)	34.2 [0.3–115.9]	31.2 [0.3–80.9]	63.5 [2.4–115.9]	0.001
VATI (cm^2^/m^2^)	30.2 [1.3–106.9]	30.6 [1.3–106.9]	24.9 [2.8–53.3]	0.827
VSR	0.91 [0.33–3.93]	1.10 [0.33–3.93]	0.46 [0.35–1.39]	0.001
Low SMI at diagnosis (yes/no)	27 / 28	22 / 20	5 / 8	0.380
Interval between 1st and 2nd CT (days)	25 [15–57]	24 [15–57]	31 [15–43]	0.506
%SMI (%)	−4.3 [−22.1–+6.4]	−4.4 [−22.1–+6.4]	−1.6 [−8.4–+5.9]	0.100
%SATI (%)	−11.5 [−52.6–+25.8]	−11.9 [−52.6–+21.8]	−11.5 [−27.5–+25.8]	0.766
%VATI (%)	−21.9 [−94.5–+286.4]	−25.3 [−94.5–+286.4]	−4.5 [−46.5–+74.5]	0.143
%VSR (%)	−5.2 [−83.8–+228.2]	−6.1 [−83.8–+228.2]	+11.4 [−51.8–+118.4]	0.312

Note

Data are presented as the median [range] or n.

Abbreviations: %SATI, percentage changes in SATI over 1 month; %SMI, percentage changes in SMI over 1 month; %VATI, percentage changes in VATI over 1 month; %VSR, percentage changes in VSR over 1 month; BMI, body mass index; BSC, best supportive care; CRT, chemo radiation therapy; RT, radiation therapy; SATI, subcutaneous adipose tissue index; SMI, skeletal muscle index; VATI, visceral adipose tissue index; VSR, visceral to subcutaneous adipose tissue area ratio.

There was no significant difference in age, TNM stage, treatment methods, and serum albumin between the male and female patients, whereas the median OS was significantly longer in males than that in females (385 vs. 215 days, *p* = 0.029). For body composition indexes, the males had significantly higher SMI (*p* = 0.004), lower SATI (*p* = 0.001), and higher VSR (*p* = 0.001) than females, whereas there was no significant difference in BMI and the incidence of low SMI at diagnosis between sexes.

The median values of %SMI, %SATI, %VATI, and %VSR in the overall cohort were −4.3%, −11.5%, −21.9%, and −5.2%, respectively, and there were no significant differences in these values according to sex.

### 3.2 Survival analysis

**FIGURE 2:**
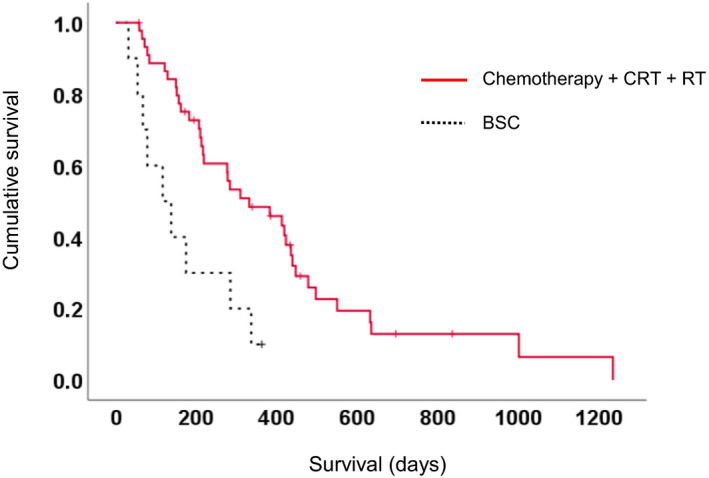
Kaplan‐Meier survival curves according to treatment methods. BSC, best supportive care; CRT, chemo radiation therapy; RT, radiation therapy

Comparison according to treatment modality showed significantly poorer survival in patients who received BSC than that in patients who underwent chemotherapy, CRT, or RT (*p* = 0.003) (Figure 2). For the analysis of the association between body composition indexes and survival, the patients were classified into the high‐ or low‐index groups based on the median baseline values of BMI (21.2 kg/m^2^), SATI (34.2 cm^2^/m^2^), VATI (30.2 cm^2^/m^2^), and VSR (0.91). As for SMI, we used the cutoffs of 42.2 cm^2^/m^2^ for males and 33.9 cm^2^/m^2^ for females.

**FIGURE 3:**
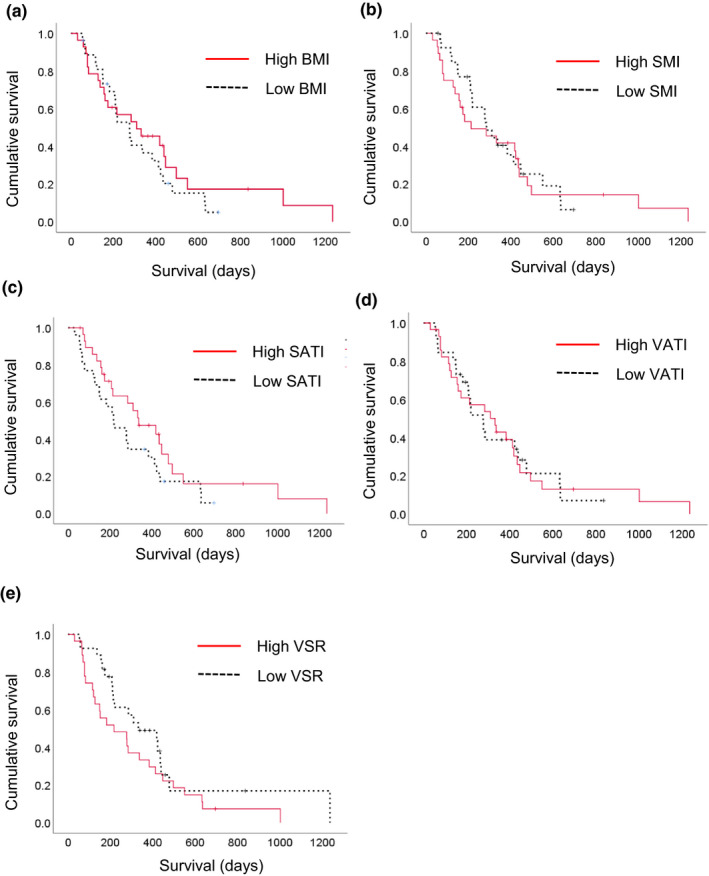
Kaplan‐Meier survival curves according to body composition indexes at baseline. (A) BMI (< or ≥21.2 kg/m^2^, *p* = 0.398), (B) SMI (< or ≥42.2 cm^2^/m^2^ for men and <or ≥33.9 cm^2^/m^2^ for women, *p* = 0.816), (C) SATI (< or ≥34.2 cm^2^/m^2^, *p* = 0.145), (D) VATI (< or ≥30.2 cm^2^/m^2^, *p* = 0.964), and (E) VSR (< or ≥0.91, *p* = 0.153). BMI, body mass index; SMI, skeletal muscle index; SATI, subcutaneous adipose tissue index; VATI, visceral adipose tissue index; VSR, visceral to subcutaneous adipose tissue area ratio

**FIGURE 4:**
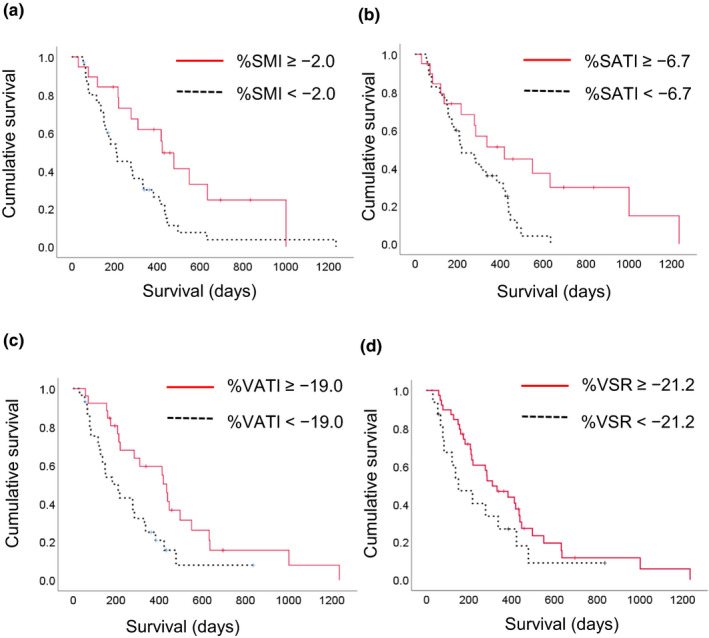
Kaplan‐Meier survival curves according to changes in body composition indexes over 1 month. (A) %SMI (< or ≥ −2.0%, *p* = 0.012), (B) %SATI (< or ≥ −6.7%, *p* = 0.018), (C) %VATI (< or ≥ −19.0%, *p* = 0.010), and (D) %VSR (< or ≥ −21.2%, *p* = 0.139). %SMI, percentage changes in skeletal muscle index over 1 month; %SATI, percentage changes in subcutaneous adipose tissue index over 1 month; %VATI, percentage changes in visceral adipose tissue index over 1 month; %VSR, percentage changes in visceral to subcutaneous adipose tissue area ratio over 1 month

In the between‐group comparison of survival, all of the body composition indexes at baseline did not have a significant impact on OS (Figure 3). As shown in Table 2, the cutoff values of %SMI, %SATI, %VATI, and %VSR were −2.0%, −6.7%, −19.0%, and −21.2%. In contrast to baseline body composition indexes, the groups with a greater decrease in the rate of change in body composition indexes including %SMI, %SATI, and %VATI had significantly worse survival than those with a non‐greater decrease (*p* = 0.012, *p* = 0.018, and *p* = 0.010, respectively, Figure 4).

**TABLE 2:** Comparison of survival according to changes in body composition indexes

Variable	Cut‐off value	Low, n	High, n	*p* value
%SMI (%)	−2.0	36	19	0.012
%SATI (%)	−6.7	35	20	0.018
%VATI (%)	−19.0	29	26	0.010
%VSR (%)	−21.2	16	39	0.139

Abbreviations: %SATI, percentage changes in subcutaneous adipose tissue index over 1 month; %SMI, percentage changes in skeletal muscle index over 1 month; %VATI, percentage changes in visceral adipose tissue index over 1 month; %VSR, percentage changes in visceral to subcutaneous adipose tissue area ratio over 1 month.

### 3.3 Correlation analysis for changes in body composition indexes

Analysis of the correlation among %SMI, %SATI, %VATI, and baseline body composition indexes showed that %SMI, %SATI, and %VATI were not significantly correlated with BMI, SMI, SATI, or VATI at baseline (Table 3). In the analyses between each of the change in body composition indexes, there was a significant correlation only between %SMI and %SATI (*r* = 0.271, *p* = 0.046).

**TABLE 3:** Correlation analyses among the body composition indexes

Variable	%SMI		%SATI		%VATI	
	r	*p* value	r	*p* value	r	*p* value
Baseline BMI	0.104	0.450	0.188	0.170	0.094	0.496
Baseline SMI	−0.090	0.516	−0.075	0.588	0.102	0.459
Baseline SATI	−0.028	0.838	0.138	0.315	0.199	0.145
Baseline VATI	−0.115	0.403	0.245	0.072	−0.138	0.314
%SMI			0.271	0.046	−0.255	0.061
%SATI	0.271	0.046			0.098	0.478
%VATI	−0.255	0.061	0.098	0.478		

Abbreviations: %SATI, percentage changes in SATI over 1 month; %SMI, percentage changes in SMI over 1 month; %VATI, percentage changes in VATI over 1 month; %VSR, percentage changes in VSR over 1 month; BMI, body mass index; SATI, subcutaneous adipose tissue index; SMI, skeletal muscle index; VATI, visceral adipose tissue index; VSR, visceral to subcutaneous adipose tissue area ratio.

### 3.4 Univariate and multivariate analyses of prognostic factors

The independent predictors of survival are shown in Table 4. In univariate analysis, sex (male: HR, 2.68; 95% CI, 1.19–6.06, *p* = 0.018), treatment modality (BSC: HR, 3.08; 95% CI, 1.41–6.72, *p* = 0.005), %SMI (< −2.0%; HR, 2.30; 95% CI, 1.18–4.47, *p* = 0.014), %SATI (< −6.7%; HR, 2.29; 95% CI, 1.14–4.61, *p* = 0.020), and %VATI (<−19.0%; HR, 2.24; 95% CI, 1.20–4.18, *p* = 0.012) were significantly associated with worse survival. Of these, male sex (HR, 2.79; 95% CI, 1.16–6.71, *p* = 0.022) and %VATI <−19.0% (HR, 2.41; 95% CI, 1.13–5.13, *p* = 0.023) were identified as independent prognostic factors in multivariate analysis (Table 3).

**TABLE 4:** Univariate and multivariate analyses of prognostic factors of overall survival

Variable	Univariate analysis		Multivariate analysis	
	HR (95% CI)	*p* value	HR (95% CI)	*p* value
Age (≥70 vs. <70 years)	1.08 (0.60–1.96)	0.798		
Sex (male vs. female)	2.68 (1.19–6.06)	0.018	2.79 (1.16–6.71)	0.022
Cancer stage (IVb vs. II, III or IVa)	1.56 (0.83–2.95)	0.161		
Primary location (head vs. body, tail)	0.99 (0.54–1.83)	0.979		
Biliary obstruction (yes vs. no)	0.81 (0.60–1.11)	0.192		
Surgical operation (yes vs. no)	0.73 (0.49–1.08)	0.117		
Treatment methods				
(BSC vs. chemotherapy, CRT or RT)	3.08 (1.41–6.72)	0.005	1.44 (0.59–3.47)	0.423
Baseline BMI (<21.2 or ≥21.2 kg/m^2^)	1.30 (0.42–1.41)	0.399		
Low SMI at diagnosis (yes vs. no)	0.93 (0.51–1.70)	0.816		
Baseline SATI (<34.2 vs. ≥34.2 cm2/m^2^)	1.56 (0.85–2.84)	0.148		
Baseline VATI (<30.2 vs. ≥30.2 cm2/m^2^)	1.01 (0.56–1.85)	0.964		
Baseline VSR (<0.91 vs. ≥0.91)	0.64 (0.35–1.18)	0.156		
%SMI (< −2.0 vs. ≥ −2.0%)	2.30 (1.18–4.47)	0.014	1.63 (0.74–3.60)	0.227
%SATI (< −6.7 vs. ≥ −6.7%)	2.29 (1.14–4.61)	0.020	1.98 (0.90–4.35)	0.089
%VATI (< −19.0 vs. ≥ −19.0%)	2.24 (1.20–4.18)	0.012	2.41 (1.13–5.13)	0.023
%VSR (< −5.2 vs. ≥ −5.2%)	1.23 (0.67–2.25)	0.499		
Albumin (<3.5 vs. ≥3.5 g/dl)	1.82 (0.67–2.47)	0.456		

Abbreviations: %SATI, percentage changes in SATI over 1 month; %SMI, percentage changes in SMI over 1 month; %VATI, percentage changes in VATI over 1 month; %VSR, percentage changes in VSR over 1 month BMI, body mass index; BSC, best supportive care; CI, confidence interval; CRT, chemo radiation therapy; HR, hazard ratio; RT, radiation therapy; SATI, subcutaneous adipose tissue index; SMI, skeletal muscle index; VATI, visceral adipose tissue index; VSR, visceral to subcutaneous adipose tissue area ratio.

Considering that the poorer survival in the BSC group might have a confounding effect in the statistical analyses, we additionally performed univariate and multivariate analyses excluding 10 patients who underwent BSC to clarify the reliability of the prognostic significance (Table S1). In univariate analysis, sex (male: HR, 2.75; 95% CI, 1.13–6.67, *p* = 0.025), %SMI (< −3.8%; HR, 2.69; 95% CI, 1.34–5.39, *p* = 0.006), %SATI (< −10.5%; HR, 2.74; 95% CI, 1.31–5.75, *p* = 0.008), and %VATI (< −19.0%; HR, 2.01; 95% CI, 1.01–4.01, *p* = 0.048) were significantly associated with worse survival. In multivariate analysis, male sex (HR, 3.34; 95% CI, 1.32–8.47, *p* = 0.011), %SMI < −3.8% (HR, 2.29; 95% CI, 1.06–4.93, *p* = 0.035), and %VATI < −19.0% (HR, 2.72; 95% CI, 1.24–5.95, *p* = 0.012) were determined as prognostic factors. Apparently, sex and %VATI remained significant risk factors for mortality, with only the addition of %SMI, even in this particular cohort.

## 4 DISCUSSION

In the current study, a high incidence (49.1%) of low SMI at diagnosis was found in patients with unresectable pancreatic cancer. Importantly, survival analysis showed a significant association between greater decreases in body composition indexes including %SMI, %SATI, and %VATI and worse survival. Only %SMI and %SATI showed significant correlation, and there was no correlation among other body composition indexes including BMI, SMI, SATI, VATI, %SMI, %SATI, and %VATI. In multivariate analysis, male sex and %VATI < −19.0% were identified as independent prognostic factors for poor prognosis.

We determined the rate of change of body composition indexes over only 1 month, which is a shorter evaluation period compared to two previous studies in patients with unresectable advanced pancreatic cancer. Dalal et al. investigated changes in body composition indexes including BMI, SMI, VATI, and SATI in 100 days.^13^ Choi et al. assessed the differences in BMI and SMI between at diagnosis and at cessation of first‐line chemotherapy due to cancer progression. The duration of assessment was not constant and probably longer than 1 month because the median progression‐free survival of first‐line chemotherapy was 3.7 months.^2^ Because most cases of advanced pancreatic cancer progress rapidly, early prediction of prognosis is particularly important. Notably, assessment during only 1 month could reveal a markedly steep decline in skeletal muscle and adipose tissue, indicated by the median values of −4.3% for %SMI, −11.5% for %SATI, and −21.9% for %VATI. Accordingly, our method of evaluation for body composition changes over 1 month is quite rational and applicable in the clinical setting for patients with unresectable pancreatic cancer.

The present study revealed that greater VAT loss, which was independent from other body component changes, had adverse effects on survival. Furthermore, VAT loss remained an independent predictor of poor prognosis even when the patients treated with BSC, who have poorer prognosis, were excluded. Our findings are consistent with those of several previous studies that demonstrated a significant correlation between higher VAT loss and worse survival in patients with resectable or unresectable pancreatic cancer.^3^, ^13^, ^18^ The majority of patients with advanced cancer irrespective of cancer types experience cachexia, defined by body weight loss caused by cancer‐related wasting both muscle and/or fat during the disease trajectory.^19^ Potential associations between fat loss and poor outcomes have also been reported in various types of advanced cancer.^20^, ^21^ Although, the exact mechanisms of adipose tissue depletion in cancer have not been elucidated to date, increased lipolysis is regarded as a main driver of fat loss in advanced cancer.^22^ Elevated activity of hormone‐sensitive lipase and adipose triglyceride lipase, which are major enzymes for breaking down triglycerides in adipose tissue, may be responsible for accelerated lipolysis.^4^, ^23^, ^24^ Other mechanisms including increased fat oxidation, decreased lipogenesis, impaired adipogenesis, and decreased lipid deposition may also contribute to fat loss in cancer.^22^ In addition, a number of pro‐inflammatory cytokines such as interleukin‐1 (IL‐1), IL‐6, tumor necrosis factor‐alpha (TNF‐α) may play a important roles in the pathological mechanisms of fat loss and cachexia.^22^, ^25^ From this point of view, some studies revealed several promising anti‐inflammatory agents for preventing cancer‐related cachexia,^26^ including non‐steroid anti‐inflammation drugs,^27^, ^28^, ^29^ which reduce tumor‐related inflammation and TNF‐α levels; IL‐6 antagonists, including ALD518^30^ and tocilizumab^31^, ^32^, ^33^; TNF‐α and IL‐6 dual‐targeting OHR/AVR118^34^; and anamorelin,^35^, ^36^ which is a ghrelin receptor agonist inhibiting pro‐inflammatory cytokines and NF‐kB. Analysis of the underlying mechanisms of VAT loss is beyond the purpose of this study, but previous epidemiological and biological studies have suggested that the detrimental effects of adipose depletion on patient outcomes in cancer may be due to potential multifactorial mechanisms.

The present study found that a greater decrease of SM was significantly associated with worse survival and that it was a significant prognostic factor in the univariate analysis. However, SM depletion was not an independent predictor of prognosis in the multivariate analysis. SM decline has been previously determined as a prognostic factor in pancreatic cancer patients undergoing various treatment.^2^, ^3^, ^4^, ^5^ The prognostic significance of SM loss both at baseline and during treatment has also been found in many other types of cancer.^37^, ^38^, ^39^ In addition, several studies reported a higher risk of chemotherapy toxicity, which was in turn associated with an increased risk of mortality, in cancer patients with low muscle mass.^5^, ^39^, ^40^ Although, these findings suggest SM depletion is a poor prognostic indicator in malignancies, VAT might be more strongly associated with prognosis than SM at least in advanced stages of pancreatic cancer. In fact, previous studies showed that loss of adipose tissue precedes loss of skeletal muscle in patients with cancer cachexia undergoing palliative chemotherapy^21^; VAT constantly decreased in advanced cancer stages, whereas SAT loss was gradually accelerated along with disease progression.^41^ These findings are consistent with our results and suggest a close relationship between VAT volume and prognosis.

The current study also showed the potential association between SAT loss and risk of mortality, as evidenced by the significant association between SAT loss and shorter survival and its significance in the univariate analysis. Although the influence of SAT on prognosis has not been established in pancreatic cancer, SAT volume at cancer diagnosis has been reported to be a poor prognostic factor in other types of cancer.^12^, ^42^ Because there are structural and functional differences between SAT and VAT,^43^ SAT may play a favorable role for prognosis via mechanisms different from that of VAT. The major biological function of SAT is energy storage. In cancer, this can protect patients from augmented energy exhaustion induced by cachexia.^3^, ^20^, ^43^ In addition, SAT has beneficial effects on lipid and glucose metabolism.^44^, ^45^ Therefore, these functions of SAT may contribute to the prolonged survival of patients with advanced pancreatic cancer, and the underlying mechanisms should be precisely elucidated to develop an effective nutritional treatment strategy for these patients.

The baseline body composition indexes including SMI, BMI, SATI, VATI, and VSR did not have significant impact on survival in the present study. In fact, the longitudinal changes of the body composition indicators were revealed as better predictors of prognosis. We defined low SMI according to the cutoff values established in a Korean large‐scale study,^2^ instead of those set by Prado et al.,^17^ in consideration of the differences in the inherent body constitution between Asian and Western people. Thus, SMI was classified avoiding a bias of the racial disparities in the standard muscle volume. Because most patients already had cancer cachexia‐related wasting of muscle and adipose tissue at the diagnosis of advanced cancer, these baseline indicators might not be helpful for determining prognoses in those patients. In contrast, evaluation of VAT loss over 1 month might enable a better prediction of prognosis and an appropriate decision for treatment; less reduction of VAT might lead to chemotherapy, whereas excessive loss of VAT might warrant induction of BSC or early cessation of chemotherapy. A prospective study is needed to validate the clinical usefulness of the short‐term assessment of VAT.

There are some limitations of the present study. The retrospective nature of the current study limits the assessment of a causal relationship between lipolysis or muscle loss and outcomes because the lack of various metabolic/nutritional parameters, adipokines, and inflammatory cytokines hamper the analyses of the underlying mechanisms of body composition changes. In addition, because the cohort enrolled was from a single tertiary center, the sample size was relatively small. The statistical results need to be carefully interpreted with respect to potential selection bias. In addition, our results may not be generalizable to other regions. Further large‐scale multicenter studies are needed to verify our findings.

Some therapeutic interventions such as exercise, nutritional support, and anti‐inflammatory therapies have been found to be beneficial for preventing progression to irreversible cachexia in cancer patients.^46^ We should be aware that cancer‐induced cachexia not only limits the performance of activities of daily living due to general fatigue and anorexia,^47^, ^48^ but is also possibly associated with high mortality. Thus, therapeutic strategies against involuntary loss of adipose tissue and skeletal muscle mass need to be established to help and improve the prognoses of patients with advanced pancreatic cancer.

## 5 CONCLUSIONS

Patients with unresectable advanced pancreatic cancer lose significant amounts of SM, VAT, and SAT over 1 month. Particularly, the rapid decline of VAT is closely associated with poor prognosis. Therefore, a short‐term assessment of VAT may be a reasonable approach to evaluate the mortality risk and make correct decisions for induction of chemotherapy or BSC and early cessation of chemotherapy for these patients.

## CONFLICT OF INTEREST

All authors declare no conflict of interest.

## ETHICAL APPROVAL STATEMENT

The study was approved by the ethics committee of the Niigata University School of Medicine (approval number 2442).

## Supporting information

Table S1

## Data Availability

The data that support the findings of this study are available from the corresponding author upon reasonable request.
