# Supplementary material for: Rapid decline in visceral adipose tissue over 1 month is associated with poor prognosis in patients with unresectable pancreatic cancer
Source: Cancer Med. 2021 May 16;10(13):4291–301. doi: 10.1002/cam4.3964 (PMC8267120; doi:10.1002/cam4.3964)
Supplement: Supplementary file 1 — Table S1 [file CAM4-10-4291-s001.docx]

**SUPPLEMENT 1**

Univariate and multivariate analyses of prognostic factors of overall survival excluding the patients undergoing best supportive care

Univariate analysis Multivariate analysis

Variable HR (95% CI) *P* value HR (95% CI) *P* value

Age (≥70 vs. <70 years) 1.28 (0.65–2.54) 0.474

Sex (male vs. female) 2.75 (1.13–6.67) 0.025 3.34 (1.32–8.47) 0.011

Cancer stage (IVb vs. II, III or IVa) 1.56 (0.75–3.26) 0.238

Treatment methods

(BSC vs. chemotherapy, CRT or RT) 1.37 (0.62–3.04) 0.443

Baseline BMI (<21.2 or ≥21.2 kg/m^2^) 1.31 (0.67–2.58) 0.431

Low SMI at diagnosis (yes vs. no) 0.93 (0.47–1.82) 0.824

Baseline SATI (<34.2 vs. ≥34.2cm^2^/m^2^) 1.72 (0.87–3.40) 0.118

Baseline VATI (<30.2 vs. ≥30.2cm^2^/m^2^) 1.08 (0.55–2.12) 0.832

Baseline VSR (<0.91 vs. ≥0.91) 0.66 (0.33–1.31) 0.230

%SMI (< −3.8 vs. ≥ −3.8%) 2.69 (1.34–5.39) 0.006 2.29 (1.06–4.93) 0.035

%SATI (< −10.5 vs. ≥ −10.5%) 2.74 (1.31–5.75) 0.008 2.02 (0.86–4.74) 0.108

%VATI (< −19.0 vs. ≥ −19.0%) 2.01 (1.01–4.01) 0.048 2.72 (1.24–5.95) 0.012

%VSR (< −5.2 vs. ≥ −5.2%) 0.78 (0.40–1.52) 0.461

Albumin (<3.5 vs. ≥3.5 g/dl) 1.13 (0.51–2.51) 0.759

Abbreviations: HR, hazard ratio; CI, confidence interval; BSC, best supportive care; CRT, chemoradiation therapy; RT, radiation therapy; BMI, body mass index; SMI, skeletal muscle index; SATI, subcutaneous adipose tissue index; VATI, visceral adipose tissue index; VSR, visceral to subcutaneous adipose tissue area ratio; %SMI, percentage changes in SMI over 1 month; %SATI, percentage changes in SATI over 1 month; %VATI, percentage changes in VATI over 1 month; %VSR, percentage changes in VSR over 1 month.
